# pH-Dependent Developmental and Behavioral Toxicity of Diethanolamine in Zebrafish (*Danio rerio*)

**DOI:** 10.3390/ijms27219935

**Published:** 2026-11-06

**Authors:** Camilo Alexandre Jablonski, Fabiola Bergozza Pereira, Julia Oliveira, Lucas Machado Bandeira, Talita Carneiro Brandão Pereira, Giovanna Medeiros Tavares de Oliveira, Luiza Wilges Kist, Vinícius Demétrio da Silva, Wesley Formentin Monteiro, William Lautert-Dutra, Marcus Seferin, Rosane Angélica Ligabue, Maurício Reis Bogo

**Affiliations:** 1 Centro de Genômica e Biologia Molecular, Escola de Ciências da Saúde e da Vida, Pontifícia Universidade Católica do Rio Grande do Sul (PUCRS), Av. Ipiranga, 6681, Porto Alegre 90619-900, RS, Brazil; camilo.aj@hotmail.com (C.A.J.); fabiola.pereira@edu.pucrs.br (F.B.P.); julia.oliveira020@edu.pucrs.br (J.O.); l.bandeira002@edu.pucrs.br (L.M.B.); talitapereira@gmail.com (T.C.B.P.); giovanna.oliveira@pucrs.br (G.M.T.d.O.); lwkist@gmail.com (L.W.K.); 2 Programa de Pós-Graduação em Medicina e Ciências da Saúde, Escola de Medicina, Pontifícia Universidade Católica do Rio Grande do Sul (PUCRS), Av. Ipiranga, 6681, Porto Alegre 90610-000, RS, Brazil; 3 Programa de Pós-Graduação em Biologia Celular e Molecular, Escola de Ciências da Saúde e da Vida, Pontifícia Universidade Católica do Rio Grande do Sul (PUCRS), Av. Ipiranga, 6681, Porto Alegre 90619-900, RS, Brazil; 4 Laboratório de Química Industrial, Escola Politécnica, Pontifícia Universidade Católica do Rio Grande do Sul (PUCRS), Av. Ipiranga, 6681, Porto Alegre 90619-900, RS, Brazil; viniciusdemetrio@gmail.com (V.D.d.S.); mseferin@outlook.com.br (M.S.); rligabue@pucrs.br (R.A.L.); 5 Programa de Pós-Graduação em Engenharia e Tecnologia de Materiais, Escola Politécnica, Pontifícia Universidade Católica do Rio Grande do Sul (PUCRS), Av. Ipiranga, 6681, Porto Alegre 90619-900, RS, Brazil; 6 Departamento de Química Inorgânica, Universidade Federal do Rio Grande do Sul (UFRGS), Av. Bento Gonçalves, 9500, Porto Alegre 91501-970, RS, Brazil; wesley.monteiro@ufrgs.br; 7 Bioinformatics Graduate Program, College of Science and Technology, Temple University, Philadelphia, PA 19140, USA; william.lautert.dutra@temple.edu; 8 Institute for Cancer Research, Fox Chase Cancer Center, Philadelphia, PA 19111, USA; 9 Cancer and Cellular Biology, Lewis Katz School of Medicine, Temple University, Philadelphia, PA 19140, USA

**Keywords:** diethanolamine (DEA), pH-mediated toxicity, cardiotoxicity, behavioral toxicity, FET, embryotoxicity

## Abstract

Amine-based compounds are used in petroleum and gas processing for CO_2_ capture and gas sweetening. Diethanolamine (DEA), a water-soluble alkaline compound, can alter exposure-medium physicochemical properties. Its industrial use and potential release into aquatic environments highlight the need to characterize its aquatic toxicity. However, data on DEA developmental toxicity remain limited, particularly regarding the distinction between intrinsic chemical toxicity and pH-mediated effects. This study investigated the developmental and behavioral toxicity of DEA in zebrafish (*Danio rerio*) under pH-adjusted and unadjusted exposure conditions to examine the contribution of exposure-medium pH to the observed effects. Acute toxicity assays determined the median lethal concentration (LC_50_), followed by sublethal assessments of spontaneous tail coiling, heart rate, morphology, and larval locomotor activity. Under pH-adjusted conditions, heart rate increased, indicating a sublethal effect on cardiac function. In contrast, unadjusted exposures caused a concentration-dependent increase in medium pH associated with adverse effects on survival, development, and behavior, despite no morphological abnormalities. For statistical analyses, individual measurements were averaged within each vessel, and vessel-level means were used to assess concentration–response relationships and estimate concentration–response trend rates. Responses differed between DEA-Adj and DEA-Unadj under their respective testing conditions, highlighting the importance of considering exposure-medium pH when assessing alkaline chemical toxicity. pH alterations caused by chemical releases may also contribute to adverse effects in aquatic organisms and should be considered when evaluating the environmental implications of DEA contamination.

## 1. Introduction

Diethanolamine (DEA) is an amine widely used in the oil and gas industry as an aqueous solvent for the removal of carbon dioxide (CO_2_) and other acid gases during refining and gas processing operations [1,2]. Although these systems are efficient, the large-scale use of DEA may pose a potential risk of environmental release via industrial effluents or operational failures, with aquatic environments as likely final recipients [3].

DEA is highly water-soluble and exhibits alkaline properties (weak base, pKb = 4.5–4.9), features that may influence its bioavailability and toxicity to aquatic organisms [4]. Variations in exposure-medium pH induced by alkaline or acidic compounds can, by themselves, affect organismal physiology and confound the interpretation of observed toxicity [5]. For example, changes in exposure-medium pH can substantially modify the apparent toxicity of ionizable compounds by altering chemical speciation and biological membrane permeability, thereby influencing toxicity outcomes independently of changes in the intrinsic mode of action of the tested substance [6,7,8]. In addition, pH may alter toxicity thresholds and redirect toxic pressure from survival endpoints toward sublethal physiological effects [9]. Reported environmental concentrations of DEA range from 0.5 to 9 µg/L in Germany and from 0.04 to 0.35 µg/L in Norway. Although reported environmental concentrations of DEA are typically several orders of magnitude lower than those used in laboratory toxicity assays, its documented presence in surface waters supports its relevance as an environmental contaminant and justifies mechanistic investigations under controlled experimental conditions [3].

Despite its extensive industrial application, information on the environmental toxicity of DEA remains limited. Most toxicological data derive from mammalian models and in vitro systems, reporting dose-dependent effects such as tissue irritation, metabolic alterations, and oxidative stress [4,10,11,12]. In contrast, ecotoxicological evidence is scarce and largely restricted to acute toxicity assays in adult fish, providing limited insight into the vulnerability of early developmental stages [13,14]. Early developmental stages represent critical windows of sensitivity to environmental contaminants, during which exposure may result not only in increased mortality, but also in persistent morphological, physiological, and behavioral disturbances with potential long-term consequences for organism survival [15,16]. In this context, zebrafish (*Danio rerio*) is a powerful and widely used experimental model for ecotoxicology and developmental toxicology, including standardized assays such as the fish embryo toxicity (FET) test [17]. However, evaluation of the environmental toxicity of DEA data addressing developmental toxicity and sublethal morphological, physiological, and behavioral effects in fish embryos and larvae remains notably underrepresented in the literature, particularly under experimental conditions that allow the distinction between intrinsic chemical toxicity and effects secondary to physicochemical properties of the exposure medium, including differences in exposure pH.

Based on these considerations, the present study aimed to evaluate the lethal and sublethal toxicity of DEA. Acute and sublethal assays were performed during the early developmental stages of zebrafish, comparing exposures conducted under pH-adjusted and unadjusted conditions and assessing endpoints related to survival, development, physiology, morphology, and behavior.

## 2. Results

### 2.1. pH

In experiments conducted with DEA-Adj, the exposure medium pH was adjusted to values close to neutrality (≈7.4) before the beginning of the experiments, in accordance with the FET test (Tables S1 and S2). Thus, animals were exposed to solutions with physiologically relevant pH throughout the experimental period, minimizing the direct influence of medium alkalinity on the evaluated endpoints.

In contrast, in experiments performed with DEA-Unadj, exposure medium pH values increased in a concentration-dependent manner during the pre-exposure phase, consistent with the alkaline nature of DEA (pH ≈ 11). Post-exposure pH was measured after 24 h of exposure, immediately before the daily renewal of solutions, which were replaced every 24 h. In assays conducted according to the FET test, pre-exposure pH ranged from 7.52 ± 0.15 in the control group to 10.10 ± 0.07 at the highest tested concentrations, whereas post-exposure pH values ranged from 7.45 ± 0.16 in the control to approximately 9.18 ± 0.04 in the most concentrated groups (Table 1). Similarly, in sublethal assays with DEA-Unadj, a progressive concentration-dependent increase in pre-exposure pH was observed, followed by a reduction after 24 h of exposure, although the pH values remained higher than those of the control group (Table 2).

### 2.2. LC_50_

The acute toxicity of DEA was evaluated under DEA-Adj and DEA-Unadj conditions using the zebrafish embryo Fish Embryo Acute Toxicity (FET) test. For DEA-Adj, test solutions were adjusted to near-neutral pH in accordance with Organization for Economic Co-operation and Development (OECD) Guideline No. 236 to ensure standardized exposure conditions (Tables S1 and S2).

Preliminary range-finding assays were conducted separately for DEA-Adj and DEA-Unadj to characterize acute toxicity under the two exposure conditions. For DEA-Adj, concentrations up to 6000 mg/L did not produce significant mortality. Therefore, three independent definitive LC_50_ assays were subsequently performed using concentrations up to 2000 mg/L. Consistently, these experiments also failed to produce ≥50% mortality in any exposure group, precluding the calculation of an LC_50_ for DEA-Adj. Because we could not estimate an LC_50_ within the tested concentration range, we adopted the highest tested concentration (2000 mg/L) as the upper limit for the sublethal toxicity assays.

In contrast, exposure to DEA-Unadj produced a clear concentration-dependent decrease in embryo survival, allowing the estimation of an LC_50_ value. The concentration–response relationship for zebrafish embryo survival after 96 h of exposure to DEA-Unadj is shown in Figure S1. The estimated LC_50_ for DEA-Unadj was 1123.8 mg/L (95% CI: 1034.3–1213.2 mg/L), based on mean survival proportions obtained from three to four independent experiments (*n* = 60–80 embryos per concentration). This LC_50_ value was subsequently used to guide the selection of exposure levels for the sublethal toxicity assays. The highest sublethal concentration was set at 300 mg/L, below one-third of the estimated LC_50_.

### 2.3. Survival

Following the determination of the LC_50_ values, sublethal exposure experiments were conducted to evaluate developmental endpoints in zebrafish embryos and larvae. Survival of embryos/larvae exposed to DEA-Adj and DEA-Unadj was monitored up to 144 h post-fertilization (hpf) (Figure S2). In both exposure approaches, survival rates remained above 95% across all groups throughout the experimental period. For both DEA-Adj (50–2000 mg/L) and DEA-Unadj (10–300 mg/L), a slight decrease in survival during the first 24 hpf was observed, followed by stabilization of the survival curves, with no clear concentration-dependent effects compared to the control group. Complementary analyses, including Dunnett-adjusted treatment comparisons and concentration–response trend analyses, supported the absence of concentration-related effects on survival (Figures S4 and S5).

### 2.4. Hatching Rate

**Figure 1:**
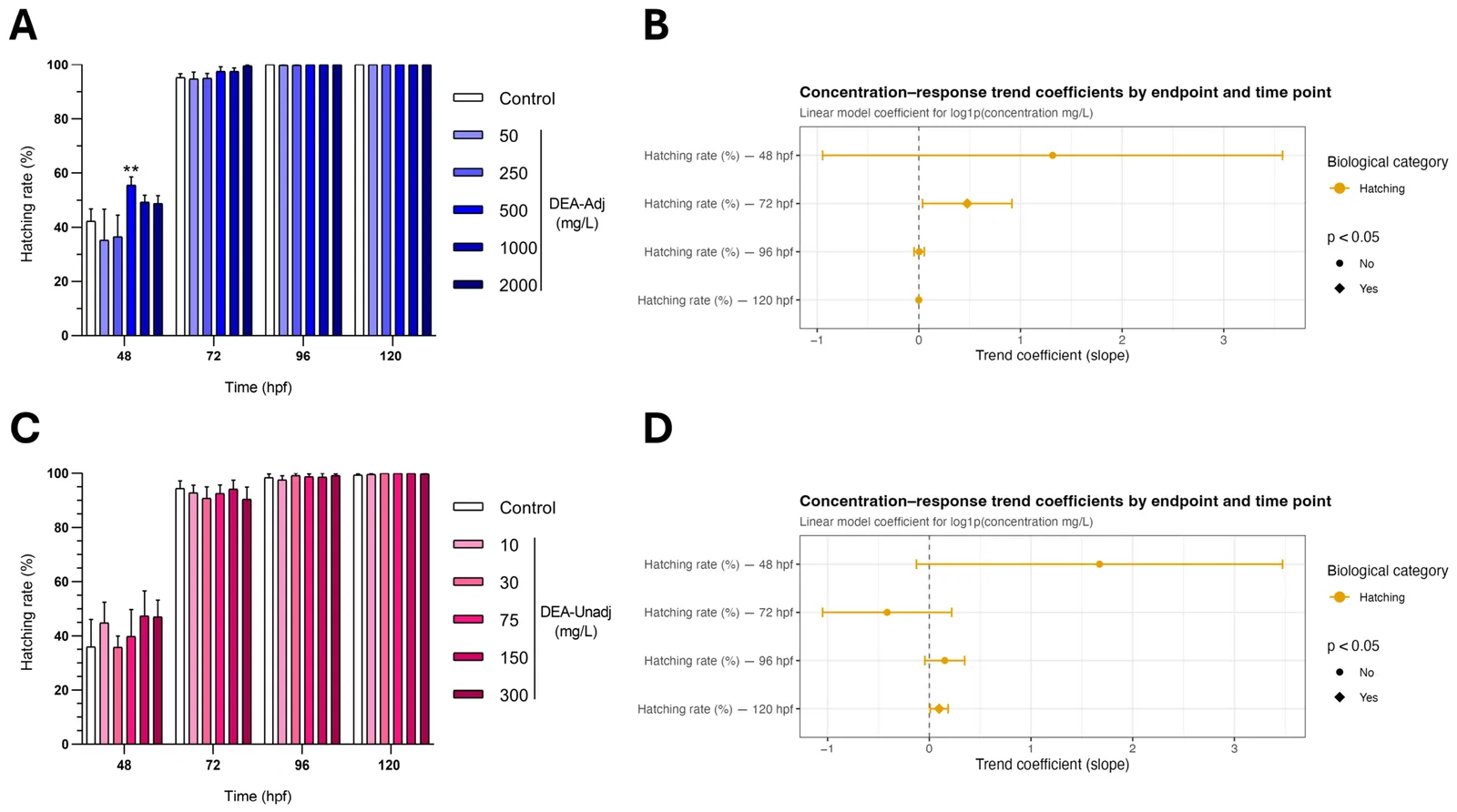
Hatching rate of zebrafish embryos exposed to DEA-Adj and DEA-Unadj at 48, 72, 96, and 120 h post-fertilization (hpf). (**A**) DEA-Adj groups compared with the control. ** *p* < 0.01. (**C**) DEA-Unadj groups compared with the control. Data are expressed as percentage hatching at each time point, and bars represent the standard error of the mean (SEM). For DEA-Adj, data represent four independent experiments, with one Petri dish per group per experiment (*n* = 4, 100 embryos/larvae assessed per Petri dish per group; 400 embryos/larvae assessed per group); for DEA-Unadj, data represent five independent experiments, with one Petri dish per group per experiment (*n* = 5, 120 embryos/larvae assessed per Petri dish; 600 embryos/larvae assessed per group). Statistical analysis was performed using two-way ANOVA followed by Dunnett’s multiple comparisons test. Asterisks indicate significant differences compared with the control (*p* = 0.002). (**B**) DEA-Adj and (**D**) DEA-Unadj concentration–response trend rate for hatching rate, calculated as the slope coefficient from a linear model fitted to vessel-level values, with log1p-transformed concentration [log1p(mg/L)] as a continuous predictor and experiment as a fixed blocking factor. Error bars indicate 95% confidence intervals. A positive coefficient indicates a higher hatching percentage with increasing DEA concentration at the specific time point.

The hatching rate was evaluated from 48 to 120 hpf in groups exposed to DEA-Adj and DEA-Unadj (Figure 1). In the DEA-Adj groups, the temporal hatching profile was generally comparable to that of the control, although a statistically significant increase was observed at 48 hpf in the 500 mg/L group. This difference was not maintained at subsequent time points (Figure 1A). In the DEA-Unadj groups, no statistically significant differences in hatching rate were detected at any evaluated time point, and final hatching rates at 120 hpf were comparable to those of the control (Figure 1C).

Complementary analyses were performed. In the DEA-Adj group, a significant positive concentration–response trend in hatching rate was detected at 72 hpf by the vessel-level linear model (model coefficient = 0.47; *p* = 0.035), and this pattern was also supported by Spearman rank correlation (rho = 0.58, *p* = 0.004; Figure 1B). In the DEA-Unadj group, a positive concentration–response trend was identified at 120 hpf by the vessel-level linear model (model coefficient = 0.097; *p* = 0.032), whereas the corresponding Spearman correlation was positive but did not reach statistical significance (rho = 0.30, *p* = 0.108; Figure 1D). The analyses of concentration–response trend rates provided an additional assessment of concentration-related patterns based on vessel-level means.

### 2.5. Spontaneous Tail Coiling (STC) and Heart Rate

**Figure 2:**
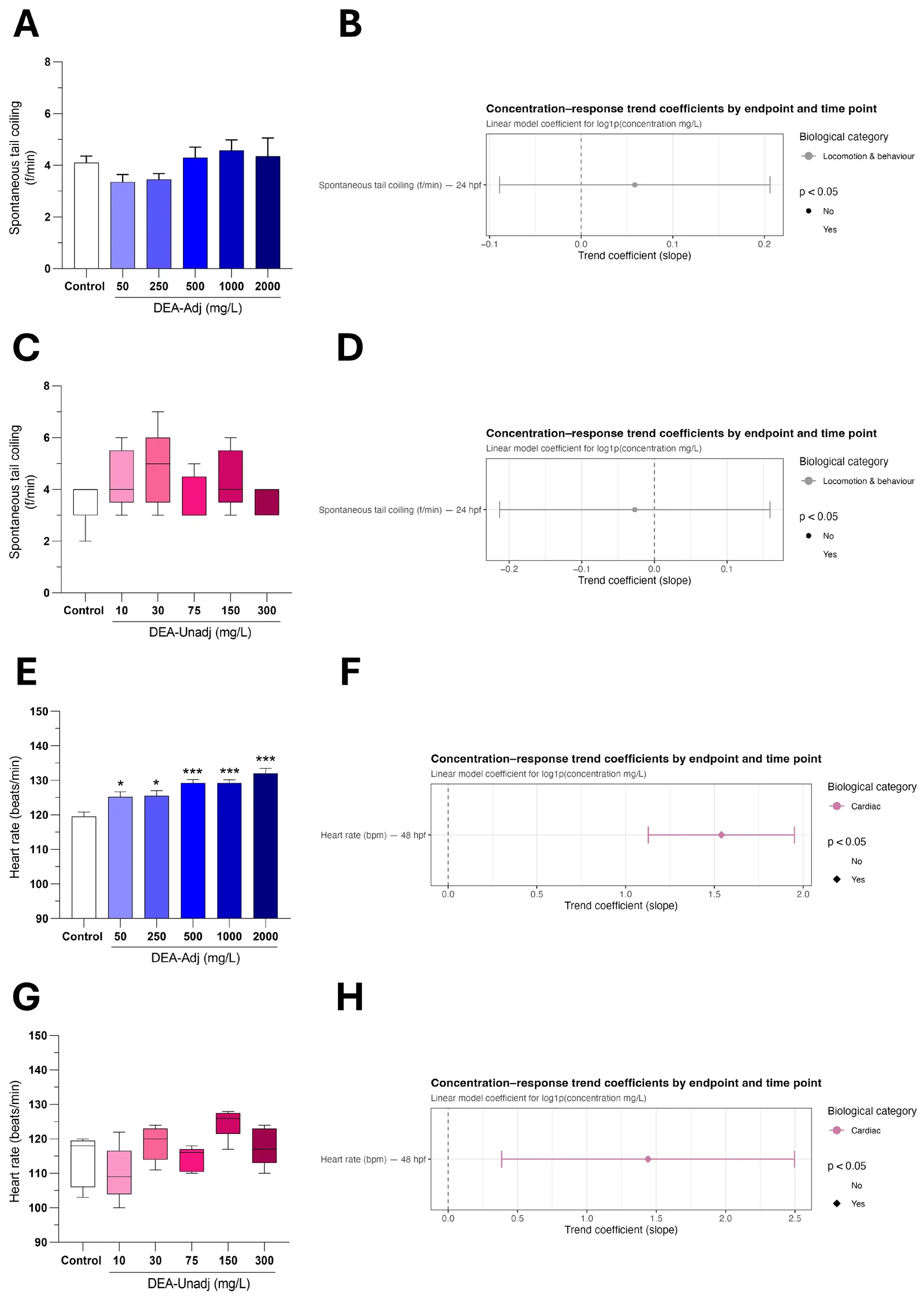
Effects of DEA-Adj and DEA-Unadj on spontaneous tail coiling (STC) and heart rate in zebrafish embryos and larvae. STC frequency at 24 h post-fertilization (hpf) following exposure to DEA-Adj (**A**) and DEA-Unadj (**C**). Data are expressed as STC frequency (f/min). Data distribution was assessed using the Shapiro–Wilk test, and statistical analysis was performed using the ANOVA test followed by Dunnett’s post hoc test and is presented as the mean ± standard error of the mean (SEM) (**A**), and Kruskal–Wallis test followed by Dunn’s post hoc test with whiskers indicating the minimum and maximum values (**C**). For DEA-Adj, data represent four independent experiments, with one vessel per group per experiment (*n* = 4, pool of 7 or 8 embryos per group); for DEA-Unadj, data represent five independent experiments, with one vessel per group per experiment (*n* = 4, pool of 7 or 8 embryos per group). Heart rate was assessed at 48 hpf in larvae exposed to DEA-Adj (**E**) and DEA-Unadj (**G**). Data are expressed as beats/min. Data distribution was assessed using the Shapiro–Wilk test, and statistical analysis was performed using the ANOVA test followed by Dunnett’s post hoc test and is presented as the mean ± standard error of the mean (**E**) and the Kruskal–Wallis test followed by Dunn’s post hoc test with whiskers indicating the minimum and maximum values (**G**). Asterisks indicate significant differences compared with the control group (* *p* < 0.05; *** *p* < 0.001). DEA-Adj (**B**) and DEA-Unadj (**D**) concentration–response trend rate for STC, and DEA-Adj (**F**) and DEA-Unadj (**H**) concentration–response trend rate for heart rate, calculated as the slope coefficient from a linear model fitted to vessel-level values, with log1p-transformed concentration [log1p(mg/L)] as a continuous predictor and experiment as a fixed blocking factor. Error bars indicate 95% confidence intervals. A positive coefficient indicates that STC and heart rate increase with DEA concentration. For DEA-Adj, data represent four independent experiments, with one vessel per group per experiment (*n* = 4, pool of 9 or 10 embryos per group); for DEA-Unadj, data represent five independent experiments, with one vessel per group per experiment (*n* = 4, pool of 9 or 10 embryos per group).

STC frequency was assessed at 24 hpf in zebrafish embryos exposed to DEA-Adj and DEA-Unadj (Figure 2). For DEA-Adj, no statistically significant differences in STC frequency were observed at concentrations ranging from 50 to 2000 mg/L when compared with the control group (Figure 2A). Similarly, exposure to DEA-Unadj at concentrations between 10 and 300 mg/L did not result in significant changes in STC frequency relative to the control (Figure 2C). These findings were supported by complementary concentration–response trend analyses (Figure 2B,D).

Heart rate was evaluated at 48 hpf in zebrafish larvae exposed to DEA-Adj and DEA-Unadj (Figure 2). In the DEA-Adj condition, heart rate was significantly increased in exposed groups compared with the control (F_(5,18)_ = 11.2, *p* ≤ 0.001) (Figure 2E). In the DEA-Unadj condition, an apparent increase in heart rate was observed at 150 mg/L, although the difference was not statistically significant (Figure 2G). Complementary concentration–response analyses indicated a positive association between DEA-Adj and DEA-Unadj concentration and increased heart rate at 48 hpf, supported by both the vessel-level trend model (DEA-Adj: model coefficient = 1.53, *p* ≤ 0.001; DEA-Unadj: model coefficient = 1.44, *p* = 0.01) and Spearman rank correlation (DEA-Adj: rho = 0.81, *p* ≤ 0.001; DEA-Unadj: rho = 0.36, *p* = 0.04) (Figure 2F,H).

### 2.6. Morphology

Larval morphological parameters were evaluated at 120 hpf following exposure to DEA-Adj and DEA-Unadj, including body length, ocular distance, and ocular surface area (Figure S3). In the DEA-Adj groups, no significant differences were observed in body length (Figure S3A), ocular distance (F_(5,18)_ = 0.2833; Figure S3C), or ocular surface area (F_(5,18)_ = 0.5511; Figure S3E) compared with the control group. Similarly, exposure to DEA-Unadj did not result in significant changes in body length (Figure S3B), ocular distance (F_(5,24)_ = 0.987; Figure S3D), or ocular surface area (F_(5,24)_ = 0.263; Figure S3F) relative to the control. Complementary Dunnett-adjusted comparisons and concentration–response trend analyses supported the absence of concentration-related effects on morphology (Figures S4 and S5).

### 2.7. Locomotor Behavior

**Figure 3:**
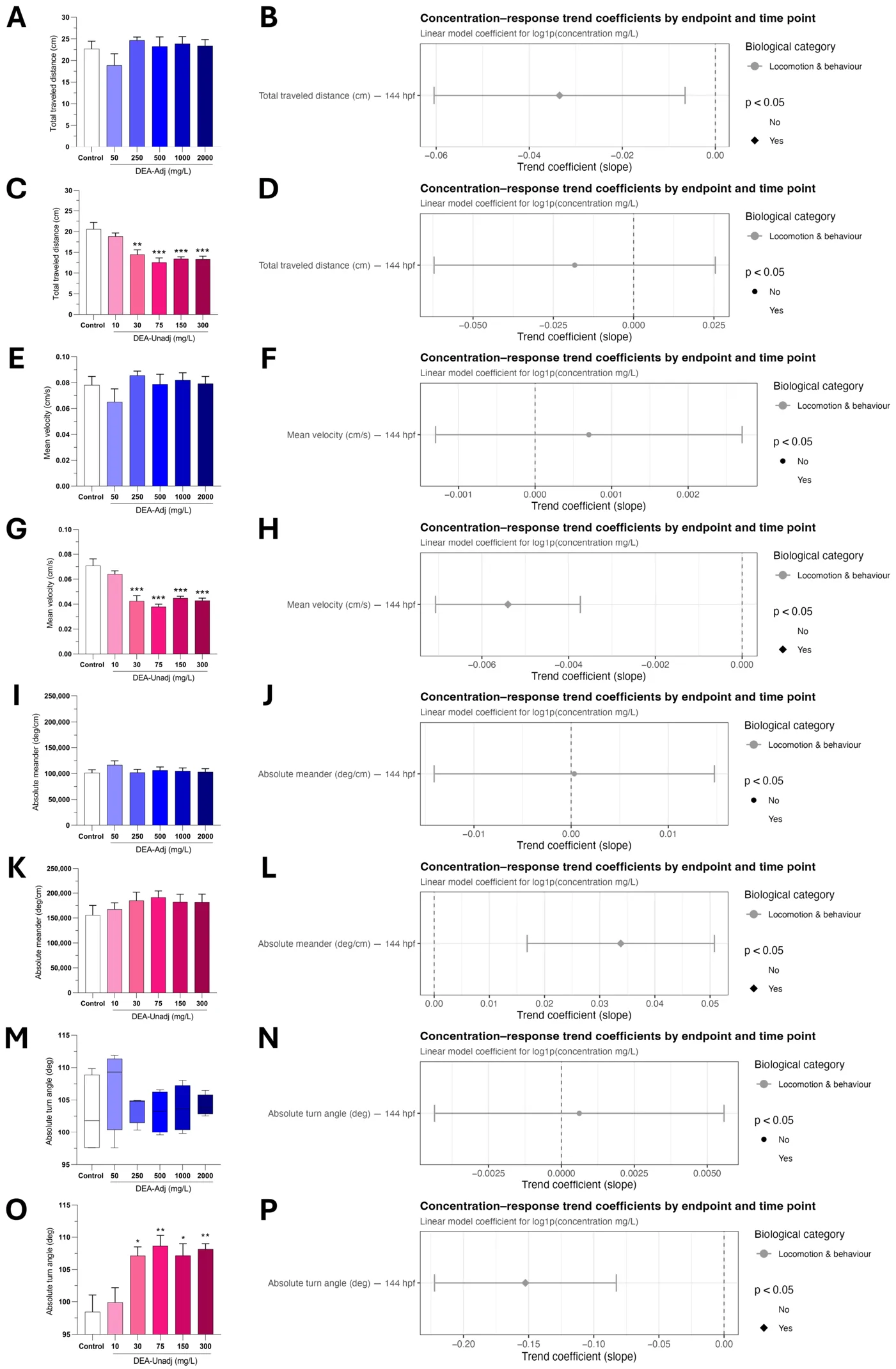
Locomotor behavior of zebrafish larvae at 144 h post-fertilization (hpf) following exposure to DEA-Adj and DEA-Unadj. (**A**,**C**) Total traveled distance. (**E**,**G**) Mean velocity. (**I**,**K**) Absolute meander. (**M**,**O**) Absolute turn angle. Panels (**A**,**E**,**I**,**M**) show DEA-Adj groups compared with the control, whereas panels (**C**,**G**,**K**,**O**) show DEA-Unadj groups compared with the control. Data distribution was assessed using the Shapiro–Wilk test, and statistical analysis was performed using the ANOVA test followed by Dunnett’s post hoc test and is presented as the mean ± standard error of the mean (SEM) (**A**,**C**,**E**,**G**,**I**,**K**,**O**), and the Kruskal–Wallis test followed by Dunn’s post hoc test, with whiskers indicating the minimum and maximum values (**M**). Asterisks indicate significant differences compared with the control group (* *p* < 0.05; ** *p* < 0.01; *** *p* < 0.001). For DEA-Adj, data represent four independent experiments, with one vessel per group per experiment (*n* = 4, pool of at least 15 larvae per group); for DEA-Unadj, data represent five independent experiments, with one vessel per group per experiment (*n* = 5, pool of at least 15 larvae per group). (**B**,**F**,**J**,**N**) DEA-Adj and (**D**,**H**,**L**,**P**) DEA-Unadj concentration–response trend rate for the locomotor behavior parameters analyzed, calculated as the slope coefficient from a linear model fitted to vessel-level values, with log1p-transformed concentration [log1p(mg/L)] as a continuous predictor and experiment as a fixed blocking factor. Error bars indicate 95% confidence intervals. A positive coefficient indicates that the locomotor behavior parameter increases with DEA concentration.

Locomotor behavior was assessed at 144 hpf following exposure to DEA-Adj and DEA-Unadj, including total traveled distance, mean velocity, absolute meander, and absolute turn angle (Figure 3). In the DEA-Adj–exposed groups, no statistically significant differences were observed in total traveled distance (F_(5,18)_ = 1.19; Figure 3A), mean velocity (F_(5,18)_ = 1.03; Figure 3E), absolute meander (F_(5,18)_ = 0.751; Figure 3I), or absolute turn angle (Figure 3M) when compared with the control group. In contrast, exposure to DEA-Unadj induced significant alterations in locomotor behavior. A significant reduction in total traveled distance was observed at 30 mg/L (*p* = 0.01), 75, 150, and 300 mg/L (*p* < 0.001) (F_(5,24)_ = 10.4; Figure 3C), along with a significant decrease in mean velocity at 30, 75, 150, and 300 mg/L (*p* < 0.001) compared with the control (F_(5,24)_ = 16.6; Figure 3G). In addition, absolute meander remained unchanged across all tested concentrations (F_(5,24)_ = 0.689; Figure 3K). Absolute turn angle was also significantly increased at 30 mg/L (*p* = 0.01), 75 mg/L (*p* = 0.003), 150 (*p* = 0.01), and 300 mg/L (*p* = 0.005) in the DEA-Unadj-exposed groups (F_(5,24)_ = 5.90; Figure 3O). Complementary vessel-level concentration–response analyses indicated positive associations between exposure concentration and locomotor activity in both the DEA-Adj and DEA-Unadj groups (Figure 3). For the DEA-Adj group, total traveled distance at 144 hpf decreased significantly with concentration (vessel-level trend model: coefficient = −0.03, *p* = 0.018; Spearman rank correlation: rho = −0.48, *p* = 0.018) (Figure 3B). For the DEA-Unadj group, mean velocity (vessel-level trend model: coefficient = −0.005, *p* ≤ 0.001; Spearman rank correlation: rho = −0.57, *p* ≤ 0.001), absolute meander (vessel-level trend model: coefficient = 0.03, *p* ≤ 0.001; Spearman rank correlation: rho = 0.31, *p* = 0.108), and absolute turn angle (vessel-level trend model: coefficient = −0.15, *p* ≤ 0.001; Spearman rank correlation: rho = −0.55, *p* ≤ 0.001) were altered at 144 hpf (Figure 3H,L,P), consistent with the concentration–response relationship analyses.

## 3. Discussion

### 3.1. LC_50_

OECD Test Guideline No. 236 provides a standardized framework for assessing acute toxicity in fish embryos under controlled exposure conditions [18]. However, when evaluating chemicals with alkaline properties, pH adjustment may help distinguish chemical-specific effects from those associated with changes in exposure-medium pH, whereas unadjusted exposures retain these changes and allow their potential contribution to the observed effects to be investigated. Accordingly, the present study compared pH-adjusted and unadjusted DEA exposures to examine how exposure-medium pH influences the developmental and behavioral responses of zebrafish (*Danio rerio*). In this context, initial acute toxicity assays conducted under pH-adjusted conditions did not result in evident mortality across the tested concentration range, which precluded the estimation of LC_50_ values. Even in preliminary assays performed at concentrations up to 6000 mg/L, no significant lethality was observed, indicating a marked attenuation of acute toxicity when exposure pH was controlled.

Based on these findings, additional experiments were conducted under unadjusted pH conditions to evaluate whether acute lethality would emerge in the absence of pH correction and to clarify the contribution of exposure medium alkalinity to the observed toxic responses. Under DEA-Unadj conditions, measurable LC_50_ values were obtained (Figure S1) whereas no LC_50_ could be determined under pH-adjusted conditions, suggesting that mortality was likely influenced by the alkaline nature of DEA and the associated elevation of exposure medium pH, as supported by the pH measurements recorded during the assays (Table 1). Early life stages of fish are highly sensitive to continuous exposure to elevated pH levels, which may disrupt essential physiological processes and contribute to lethality, as demonstrated in experimental studies evaluating pH effects in fish embryos and larvae [19].

However, the relatively high LC_50_ values obtained under unadjusted conditions suggest a limited apparent sensitivity of zebrafish embryos to DEA itself. This interpretation is consistent with acute toxicity data reported for other freshwater fish species, indicating that DEA exhibits low to moderate acute toxicity in fish, with 96 h LC_50_ values in the high mg/L range, such as approximately 1460 mg/L for *Pimephales promelas* [13] and around 460 mg/L for *Oncorhynchus mykiss* [14]. These values are comparable in magnitude to those observed in the present study and support the interpretation that relatively high concentrations of DEA are generally required to induce acute lethality in fish. Furthermore, comparative assessments have shown that lethality-based endpoints in embryonic stages tend to be less sensitive than those observed in larval or juvenile stages [20], which may further contribute to elevated LC_50_ estimates in FET assays. In addition, LC_50_ values derived from the FET assay may be influenced by physicochemical confounding factors, such as deviations from neutral pH, potentially leading to under- or overestimation of effect concentrations [21]. These findings indicate that the LC_50_ values obtained in the present study should be interpreted within the context of both the intrinsic properties of DEA and the physicochemical characteristics of the exposure medium.

### 3.2. Survival and Hatching Rate in Sublethal Exposure Assays

Survival remained above 95% in all groups exposed to DEA-Adj and DEA-Unadj throughout the experimental period (Figure S2). Survival rates remained within the expected range even in groups where exposure medium pH increased under unadjusted conditions (Table 2), indicating that the observed physicochemical changes did not compromise overall viability at the sublethal concentrations tested. This pattern was further supported by vessel-level analyses, with neither Dunnett-adjusted treatment contrasts nor concentration–response trend analyses indicating a significant effect on survival (Figures S4 and S5). This pattern is consistent with sublethal exposure designs based on LC_50_-derived concentrations, in which mortality is expected to be minimal to allow the investigation of more sensitive developmental endpoints [22]. Previous zebrafish embryo toxicity studies and methodological reviews indicate that mortality is a relatively insensitive endpoint under sublethal exposure conditions and that the absence of lethality does not preclude the occurrence of physiological, morphological, or behavioral disturbances at later developmental stages [20,23].

Hatching dynamics were comparable to the control. In the DEA-Adj groups, a statistically significant increase in hatching rate was observed at 48 hpf in the 500 mg/L group (Figure 1A); however, this effect was not sustained in later time points, and final hatching rates were like those of the control. The complementary vessel-level analyses also indicated positive associations between concentration and hatching in both the DEA-Adj and DEA-Unadj groups (Figure 1B,D). Nevertheless, the lack of persistence of the observed effect across subsequent time points suggests that this concentration-related pattern should be interpreted cautiously, particularly given that zebrafish hatching occurs asynchronously within a clutch and may be influenced by intrinsic developmental variability. These factors may limit the sensitivity of hatching rate as a precise indicator of developmental stage [24]. In the DEA-Unadj groups, no significant effects on hatching were detected at any evaluated time point, further supporting the absence of a consistent alteration in hatching dynamics under sublethal exposure conditions (Figure 1C).

### 3.3. Spontaneous Tail Coiling (STC)

Spontaneous tail coiling activity (24 hpf) was not significantly affected by DEA exposure under either DEA-Adj or DEA-Unadj conditions. In the DEA-Adj-exposed embryos (50–2000 mg/L), STC frequencies were comparable to the controls, with overlapping distributions and no concentration-dependent pattern (Figure 2A). Likewise, DEA-Unadj exposure (10–300 mg/L) did not result in consistent changes in median STC frequency despite some individual variability (Figure 2C). These findings were further supported by the concentration–response trend analyses, which did not indicate a significant concentration-related change in STC frequency under either exposure condition. STC is a sensitive early neurodevelopmental endpoint reflecting the maturation of spinal motor circuits and early motor neuron activity [25]; however, the absence of significant effects indicates that neither DEA itself nor the concentration-dependent alkalinization observed under unadjusted conditions (Table 2) was sufficient to disrupt spontaneous motor output at this developmental stage.

### 3.4. Heart Rate

Heart rate (48 hpf) emerged as a sensitive physiological endpoint in the present study. Under DEA-Adj conditions, significant increases were observed across all tested concentrations (Figure 2E). Because exposure pH was maintained near neutrality (Table S2) and physicochemical conditions were comparable to those of the control at lower concentrations, this consistent tachycardic response suggests a predominant direct sublethal effect of DEA rather than an effect primarily driven by changes in medium properties. Although conductivity increased at higher DEA-Adj concentrations (Table S4), likely reflecting the increased ionic content associated with pH adjustment using HCl, tachycardia was also observed at lower concentrations, where conductivity remained close to the control values. This suggests that the cardiac response is unlikely to be explained solely by increased ionic strength. Under DEA-Unadj conditions, no statistically significant differences from the control were observed (Figure 2G). The basis for this response pattern remains unclear. Nevertheless, complementary vessel-level concentration–response analyses identified positive associations between DEA concentration and heart rate under both the DEA-Adj and DEA-Unadj conditions (Figure 2F,H), providing complementary evidence of a concentration-related cardiac response. As the DEA-Unadj exposures were accompanied by concentration-dependent increases in medium pH (Table 2), the observed cardiac profile may reflect an interaction between chemical exposure and alkalinity-related stress, since environmental pH influences ionic regulation in fish embryos, thereby modulating membrane excitability and associated physiological processes [26]. Taken together, these findings indicate that multiple physiological mechanisms may contribute to the observed alterations in heart rate. Evidence from rodent studies demonstrates that subchronic exposure to diethanolamine is associated with cardiac toxicity, including increased heart weight and myocardial degeneration or necrosis, supporting the heart as a potential target organ of DEA toxicity [10]. In the present study, pH adjustment with HCl under DEA-Adj conditions may have promoted partial protonation of DEA and the ionic composition of the exposure medium, potentially influencing DEA bioavailability and physiological responses [27,28]. Since neither the speciation of DEA, internal DEA levels, nor disturbances in ionic or acid–base homeostasis were directly evaluated in this study, their potential roles in the observed effects remain to be investigated. Disturbances in ionic or acid–base homeostasis are also known to influence cardiac function in vertebrates [29]. Experimental studies in zebrafish further indicate that tachycardia may represent a stress-related physiological response mediated by autonomic regulation [30,31]. Accordingly, the consistent tachycardia observed under DEA-Adj conditions may reflect a direct sublethal effect of DEA under near-neutral pH conditions, whereas under DEA-Unadj conditions, medium alkalinity appears to act as a relevant modulatory factor. Further experiments are needed to determine the mechanisms underlying these responses. Finally, direct quantitative comparisons between DEA-Adj and DEA-Unadj conditions should be interpreted with caution, as different concentration ranges were applied in each experimental design.

### 3.5. Morphology

Exposure to DEA under both pH-adjusted and unadjusted conditions did not induce significant alterations in larval morphology at 120 hpf, as evidenced by the absence of changes in body length, ocular distance, and ocular surface area across all tested concentrations (Figure S3). These findings were further supported by vessel-level analyses, with neither Dunnett-adjusted treatment contrasts nor concentration–response trend analyses indicating concentration-related effects on the evaluated morphological parameters (Figures S4 and S5). The concordant lack of effects in both the DEA-Adj and DEA-Unadj groups indicates that DEA exposure did not impair overall growth or structural development during early life stages.

Morphological endpoints are widely used in zebrafish toxicology, and quantitative morphometric measurements can improve the objectivity and reproducibility of developmental assessments compared with qualitative observations alone [24,32,33]. Therefore, the absence of morphological effects suggests that DEA does not markedly interfere with these processes within the concentration ranges evaluated. These findings suggest that effects observed in the present study occurred in the absence of overt morphological abnormalities or gross developmental malformations.

### 3.6. Locomotor Behavior

The evaluation of locomotor behavior at 144 hpf revealed distinct response patterns between pH-adjusted and unadjusted DEA exposures (Figure 3), providing additional insight into the sublethal neurobehavioral consequences of DEA during early development. No significant behavioral alterations were observed under DEA-Adj conditions, whereas DEA-Unadj exposure induced a consistent pattern of locomotor alterations involving both activity-related and trajectory-related parameters, suggesting impaired swimming performance. Complementary concentration–response trend rate analyses identified positive concentration–response associations for total traveled distance (Figure 3B) in DEA-Adj, and mean velocity (Figure 3H), absolute meander (Figure 3L), and absolute turn angle (Figure 3P) in the DEA-Unadj groups, providing additional evidence of concentration-related behavioral responses.

Under DEA-Adj conditions, no significant changes were detected in total traveled distance, mean velocity, absolute meander, or absolute turn angle (Figure 3A,E,I,M), indicating the preservation of locomotor capacity and swimming performance. However, trend analysis identified a reduction in the total distance traveled. In contrast, DEA-Unadj exposure produced a coherent pattern of locomotor disruption, characterized by significant reductions in total traveled distance (Figure 3C) and mean velocity (Figure 3G), together with increases in absolute turn angle (Figure 3O). Decreased distance and velocity are indicative of hypoactivity, while increased turn angle reflects impaired swimming efficiency and loss of directional control [34]. The simultaneous alteration of these endpoints supports the interpretation of a genuine neurobehavioral disturbance, suggestive of disrupted sensorimotor integration and motor coordination rather than nonspecific variability.

From an ecotoxicological perspective, locomotor behavior represents an integrative endpoint closely linked to ecologically relevant functions such as foraging efficiency, predator avoidance, and habitat exploration [35]. Accordingly, the impairments observed under DEA-Unadj conditions may have important implications for larval fitness, even in the absence of overt morphological defects or increased mortality.

Under DEA-Adj conditions, pH adjustment appears to modulate locomotor responses in exposed larvae, potentially preventing detectable impairment. Similar pH-dependent modulation of locomotor behavior has been reported in zebrafish larvae exposed to pharmacological agents, indicating that small variations in environmental pH can influence behavioral outcomes without necessarily inducing overt locomotor deficits [36]. In contrast, the pronounced effects observed under DEA-Unadj conditions likely arise from the combined action of intrinsic DEA toxicity and pH-related physicochemical stress, which may exacerbate disturbances in ionic regulation, neuronal excitability, and neuromuscular function [37]. To further investigate the mechanisms underlying these findings, future studies incorporating biochemical markers, such as acetylcholinesterase activity, would be valuable to explore the potential involvement of cholinergic signaling and neuromuscular function in the observed behavioral alterations.

An important consideration when interpreting the differences between DEA-Adj and DEA-Unadj is that pH adjustment with HCl alters the acid–base equilibrium of DEA, increasing the proportion of protonated DEA and introducing chloride ions into the exposure medium. Therefore, the adjusted condition does not represent exposure exclusively to unprotonated DEA. Because the experimental design did not independently distinguish the effects of exposure-medium pH from those associated with changes in DEA speciation, the observed biological responses cannot be attributed exclusively to either factor. Accordingly, the differences between the two exposure conditions should be interpreted as reflecting the combined influence of exposure-medium pH and the associated acid–base speciation of DEA. This consideration highlights the importance of accounting for both factors when interpreting the developmental, physiological, and behavioral effects observed in aquatic toxicity assays involving alkaline compounds. In addition, the absence of additional controls limits the distinction between the effects of pH, ionic strength, and DEA speciation on the observed biological responses. The lack of an additional pH control represents a limitation of the present study.

## 4. Materials and Methods

### 4.1. Diethanolamine

Diethanolamine (≥99%, Sigma-Aldrich, St. Louis, MO, USA) was obtained from Sigma-Aldrich (St. Louis, MO, USA) and used as the test compound under pH-adjusted (DEA-Adj) and unadjusted (DEA-Unadj) conditions. DEA is a low-molecular-weight alkanolamine that is highly miscible in water, allowing rapid and homogeneous dissolution in aqueous media. DEA was weighed using an analytical balance and freshly diluted in water from the zebrafish maintenance system of the Centro de Modelos Biológicos Experimentais (CeMBE), Pontifícia Universidade Católica do Rio Grande do Sul (PUCRS), Brazil, to yield the stock solution. For the LC_50_ determination assays, stock solutions of 2000 mg/L (DEA-Adj) and 4000 mg/L (DEA-Unadj) were prepared, whereas for the sublethal exposure experiments, stock solutions of 2000 mg/L (DEA-Adj) and 300 mg/L (DEA-Unadj) were used. For DEA-Adj, the pH of the stock solution was adjusted to near-neutral values (≈7.0–7.4) using 6 M hydrochloric acid (HCl) in accordance with Fish Embryo Toxicity (FET) Guideline No. 236 [18], whereas for DEA-Unadj, the pH was not adjusted and reflected the intrinsic alkalinity of DEA. In all experimental conditions, the pH and conductivity of the exposure media were measured daily (Table 1, Table 2 and Tables S1–S4). The stock solution was prepared one day before the beginning of the experiments, briefly homogenized under magnetic stirring, sealed with plastic film to prevent evaporation, and stored in a BOD incubator throughout the experimental period (26–28.5 ± 1 °C). At the beginning of the experiment and before each daily preparation, the stock solution was again subjected to magnetic stirring for ≥1 min. For the LC_50_ determination assays, working solutions were prepared in 100 mL glass bottles with screw caps, whereas for sublethal exposure experiments, working solutions were prepared in volumetric flasks. In both cases, working solutions were obtained by dilution of the stock solution in system water and manually homogenized. Exposure solutions were renewed every 24 h, characterizing a semi-static exposure system. The same procedure was applied to both the LC_50_ determination assays and sublethal exposure experiments.

### 4.2. Zebrafish Husbandry, Reproduction, and Embryo Collection

Adult zebrafish (*Danio rerio*, AB strain) were maintained in continuous-flow water systems (Zebtec–Tecniplast^®^, Buguggiate, VA, Italy) under automatically controlled physicochemical conditions (27 ± 1 °C; pH 7–8; conductivity 500–800 µS; 14:10 h light:dark cycle) following standard zebrafish husbandry procedures [38]. Fish were fed twice daily with commercial flake food (TetraMin Tropical Flake, Tetra) supplemented with *Artemia* sp. during the evening feeding. For breeding, males and females were separated at a ratio of 2:1, respectively, and placed in small breeding tanks at the end of the day. In the early hours of the following morning, the physical divider was removed to allow mating for 30–60 min. Fertilized embryos were then collected, cleaned, and used for subsequent experiments. All procedures described in this study were previously approved by the Institutional Animal Care and Use Committee of Pontifícia Universidade Católica do Rio Grande do Sul (CEUA/PUCRS, No. 11123, approved on 25 October 2022).

### 4.3. Median Lethal Concentration (LC_50_)

Acute toxicity assays were conducted using the FET protocol described in OECD Test Guideline No. 236 [18], with pH adjustment under DEA-Adj conditions and the intentional omission of pH adjustment under DEA-Unadj conditions to investigate toxicity in exposure media reflecting the intrinsic alkalinity of DEA. Working solutions were prepared by serial dilution of the corresponding stock solutions described in Section 4.1. For DEA-Adj, preliminary range-finding tests were conducted using concentrations up to 6000 mg/L to identify a concentration range capable of inducing embryo lethality. Based on these preliminary assays, working concentrations of 125, 250, 500, 1000, and 2000 mg/L were selected for the definitive LC_50_ test. Exposure solutions were adjusted to near-neutral pH prior to the start of the experiment, in accordance with the FET guideline. For DEA-Unadj, embryos were exposed to working concentrations of 300, 500, 700, 900, 1100, 1300, 1600, 2000, 2400, and 2800 mg/L, obtained by serial dilution, without pH adjustment. Zebrafish fertilized eggs up to 1.5 h post-fertilization (hpf) were individually selected under a stereomicroscope (SZ40 stereo zoom microscope, Olympus^®^, Hachioji-shi, Tokyo, Japan) to ensure normal fertilization and developmental stage and allocated to 24-well plates, with 2 mL of exposure solution per well. Embryos were exposed for 96 h under semi-static conditions, with the daily renewal of test solutions, in an incubator maintained at 26 ± 1 °C and a 14:10 h light:dark photoperiod. The pH and conductivity of the exposure media were measured daily at solution renewal, both in freshly prepared solutions intended for the next exposure interval and in solutions sampled at the end of each 24 h exposure period (Table 1, Table 2 and Tables S1–S4). Embryo lethality was assessed every 24 h using a stereomicroscope (SZ40 stereo zoom microscope, Olympus^®^, Hachioji-shi, Tokyo, Japan) according to the four apical endpoints defined by OECD Guideline No. 236: coagulation of fertilized eggs, absence of somite formation, lack of tail detachment, and absence of heartbeat. Mortality data obtained at 96 h post-fertilization were used to estimate the LC_50_ value using the Toxicity Relationship Analysis Program (TRAP, U.S. EPA; version 1.30a).

### 4.4. Sublethal Exposures: Experimental Design

**Figure 4:**
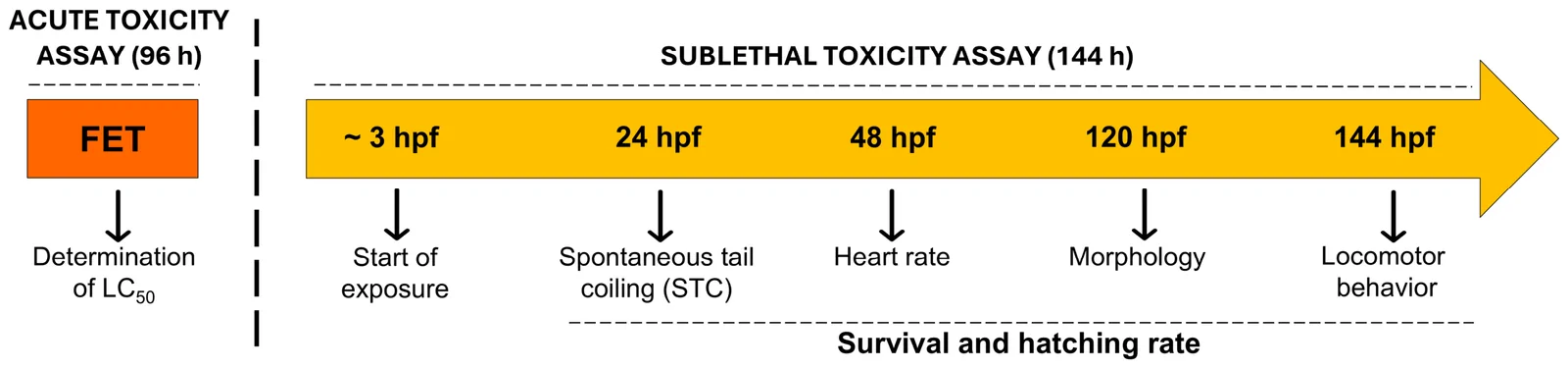
Experimental design for diethanolamine (DEA) exposure. Healthy embryos showing normal cleavage patterns were selected under a stereomicroscope and exposed to DEA diluted in system water. The Fish Embryo Toxicity (FET) test (OECD No. 236; from ≤1.5 to 96 hpf) was used to determine the LC_50_, whereas the sublethal exposure protocol lasted from approximately 3 to 144 hpf (approximately 6 days). Both protocols were conducted under semi-static conditions, with daily renewal of freshly prepared test solutions. This experimental design was used for both pH-adjusted (DEA-Adj) and unadjusted (DEA-Unadj) exposure conditions. Spontaneous tail coiling (STC) was assessed at 24 hpf, heart rate at 48 hpf, morphological endpoints at 120 hpf, and locomotor behavior at 144 hpf. Survival and hatching rates were monitored throughout the exposure period. [hpf: hours post-fertilization; LC_50_: median lethal concentration].

Sublethal exposure assays were conducted separately for DEA-Adj and DEA-Unadj, and the experimental design (Figure 4) was defined according to the outcomes of the LC_50_ and based on a previously published sublethal toxicity study [39]. For DEA-Adj, since no LC_50_ value could be estimated within the tested concentration range in the acute exposure experiments, sublethal concentrations were established based on the highest concentration evaluated in the LC_50_ assays (2000 mg/L). Accordingly, embryos were exposed to DEA-Adj at concentrations of 50, 250, 500, 1000, and 2000 mg/L, in addition to a control group (system water). Exposure solutions were prepared by serial dilution of the corresponding stock solutions described in Section 4.1, following the same preparation procedures. The exposure solutions were prepared under pH-adjusted conditions, as described in Section 4.1.

For DEA-Unadj, the estimated LC_50_ obtained from the acute toxicity assays was used to guide the selection of sublethal concentrations. Sublethal concentrations were selected to remain at or below one-third of the LC_50_ value [22], resulting in a maximum concentration of 300 mg/L. Thus, embryos were exposed to DEA-Unadj at concentrations of 10, 30, 75, 150, and 300 mg/L, along with a control group (system water). Exposure solutions were obtained by serial dilution of the corresponding stock solution described in Section 4.1. In these assays, no pH adjustment was performed, and exposure solutions reflected the intrinsic alkalinity of DEA.

Freshly fertilized eggs were collected and selected under a stereomicroscope (SZ40 stereo zoom microscope, Olympus^®^, Hachioji-shi, Tokyo, Japan), and only normally developing eggs up to 3 hpf were used. Animals were randomly assigned to the experimental group to ensure an unbiased distribution among treatments and exposed for 6 days, following the protocol described by [40]. Exposure solutions were renewed every 24 h to maintain chemical stability and water quality. Embryos/larvae were maintained in a BOD incubator under controlled conditions (28.5 ± 1 °C; 14:10 h light:dark photoperiod), which represent standard conditions for zebrafish embryonic and larval development. The pH of the exposure media was measured immediately before exposure initiation and after each 24-h exposure interval, before solution renewal.

Survival and hatching were recorded daily using a stereomicroscope (SZ40 stereo zoom microscope, Olympus^®^, Hachioji-shi, Tokyo, Japan) throughout the exposure period. Spontaneous tail coiling (STC) was evaluated at 24 hpf as an early functional indicator of neuromuscular development, as spontaneous movements emerge during early embryogenesis [41]; heart rate was assessed at 48 hpf when cardiac activity is well-established and can be used to evaluate cardiovascular function during embryonic development [42]); larval morphology was analyzed at 120 hpf, a standard time point for evaluating developmental toxicity and identifying structural abnormalities in zebrafish embryos and larvae [18]); and locomotor behavior was evaluated at 144 hpf, when larval swimming activity and behavioral patterns can be evaluated and show developmental changes [35]. These time-points were selected according to the developmental stage of zebrafish and the biological relevance of each endpoint [40]. Survival was monitored daily to confirm sublethal exposure conditions. Sublethal exposure assays comprised four independent experiments for DEA-Adj and five independent experiments for DEA-Unadj. The number of embryos/larvae used in the survival and hatching assessments was determined to enable subsequent analyses, thereby minimizing additional animal use in accordance with the 3Rs principles and the ARRIVE guidelines.

#### 4.4.1. Embryonic Endpoints: Spontaneous Tail Coiling (STC) and Heart Rate

At 24 hpf, spontaneous tail coiling (STC) was assessed to evaluate early motor activity during zebrafish embryonic development. Embryos were removed from the incubator and acclimatized for 1 min at room temperature (approximately 24–25 °C). STC activity was recorded using a smartphone camera coupled to a stereomicroscope (DI-106T trinocular stereo microscope, Digilab, Piracicaba, São Paulo, Brazil) at 60 frames per second. After the acclimation period, 10 embryos per group were simultaneously filmed for 1 min for subsequent offline quantification of tail coiling events (*n* = 10/group/experiment; 4 independent experiments for DEA-Adj and 5 independent experiments for DEA-Unadj).

At 48 hpf, heart rate was assessed to evaluate cardiac function during embryonic development. Before assessment, embryos were allowed to acclimatize at room temperature (approximately 24–25 °C) for 1 min before heart rate assessment. Cardiac activity was evaluated under a stereomicroscope (SZ40 stereo zoom microscope, Olympus^®^, Hachioji-shi, Tokyo, Japan; DI-106T trinocular stereo microscope, Digilab, Piracicaba, São Paulo, Brazil) by direct visual counting of heartbeats. For each embryo, heartbeats were counted in three independent 10-s intervals, and the mean value was subsequently converted and expressed as beats per minute (bpm) (*n* = 8/group/experiment; 4 independent experiments for DEA-Adj and 5 independent experiments for DEA-Unadj).

#### 4.4.2. Morphological Evaluation

At 120 hpf, to identify potential developmental abnormalities, larvae without visible morphological or locomotor deformities were selected for image acquisition using a stereomicroscope equipped with a digital camera (SMZ1500 Stereo Microscope, Nikon^®^, Melville, NY, USA) at 3× magnification. Morphological analyses were performed in dorsal view based on the following parameters: body length (mm), ocular distance (µm), and ocular surface area (µm^2^). (*n* = 6/group/experiment; 4 independent experiments for DEA-Adj and 5 independent experiments for DEA-Unadj). All measurements were obtained from calibrated dorsal images using ImageJ software (version 1.54s; National Institutes of Health, Bethesda, MD, USA; https://imagej.net/ij/download.html, accessed on 4 November 2026).

#### 4.4.3. Locomotor Behavior

At 144 hpf, to characterize larval swimming performance, larval locomotor behavior was evaluated (*n* = 18/group/experiment; 4 experiments for DEA-Adj; 5 experiments for DEA-Unadj). Only larvae without visible morphological abnormalities or evident locomotor impairments were selected for analysis. Larvae were individually placed in 24-well plates, with nine wells used simultaneously per recording (one larva per well), and allowed to acclimate for 1 min at room temperature (approximately 25–26 °C). Two videos were recorded per experiment, totaling 18 larvae analyzed per group in each experiment. Locomotor activity was recorded for 5 min at 25 frames/s, allowing the quantification of the behavioral parameters investigated in this study, using a high-resolution digital camera (AltaVision^®^, Basler ace, model scA1300-60gc, Ahrensburg, Schleswig-Holstein, Germany) coupled to EthoVision XT software 18 (Noldus Information Technology, Wageningen, The Netherlands). The following locomotor parameters were quantified: total traveled distance (cm), mean velocity (cm/s), absolute meander (deg/cm), and absolute turn angle (deg).

### 4.5. Statistical Analyses

Statistical analyses based on vessel-level means were used to evaluate concentration–response relationships and estimate concentration–response trend rates across the evaluated endpoints. First, statistical analyses were performed to evaluate concentration–response relationships with GraphPad Prism (version 8.0.2; GraphPad Software, San Diego, CA, USA). The Shapiro–Wilk test was used to assess normality for all in vivo endpoints except survival and hatching. Survival curves were compared using the log-rank (Mantel–Cox) test. Hatching rates were evaluated by two-way analysis of variance (ANOVA), followed by Dunnett’s multiple-comparison test, and are reported as mean ± standard error of the mean (SEM). For datasets that did not meet the parametric assumptions, the Kruskal–Wallis test followed by Dunn’s post hoc test was employed, with results expressed as medians and interquartile ranges. Parametric datasets were analyzed by one-way ANOVA followed by Dunnett’s post hoc test and are presented as mean ± standard error of the mean (SEM). Statistical significance was established at *p* ≤ 0.05.

Complementary analyses were performed to estimate the concentration–response trend rate across the evaluated endpoints, as previously described [43]. Four or five independent experiments were carried out for all in vivo endpoints. In each experiment, one vessel was used for each treatment. Before statistical analysis, measurements from individual organisms within the same vessel were averaged, generating one vessel-level observation for each endpoint, time point, concentration, and experiment. This approach was adopted to minimize the potential effects of pseudoreplication [44,45]. Between-experiment variation was incorporated into the statistical models by specifying experiment as a fixed blocking factor. Differences between each treatment and the control were assessed through Dunnett-adjusted contrasts [46] based on linear models fitted to the vessel-level data. Concentration-dependent monotonic responses were further examined using linear regression models in which log1p-transformed concentration (log1p[mg/L]) was entered as a continuous predictor, while experiment was retained as a blocking factor. Concentration–response trends were tested for each endpoint and time point using linear models of the form vessel mean ~ log(1 + concentration) + experiment [47]. The log1p transformation was selected to facilitate linear modelling of the concentration–response relationship while allowing the zero-concentration control to be included in the analysis. For these models, the estimated slope was considered the primary measure of effect. Spearman rank correlation analyses were additionally performed as a non-parametric assessment of the association between concentration and vessel-level endpoint values. All analyses were conducted in R (version 4.4.1) using the dplyr, ggplot2, broom, and emmeans packages.

## 5. Conclusions

To the best of our knowledge, this is the first study to investigate the developmental toxicity of diethanolamine (DEA) in zebrafish early life stages by explicitly comparing pH-adjusted and unadjusted exposure conditions and integrating lethal and sublethal endpoints. Under pH-adjusted conditions, DEA exposure did not induce mortality, morphological alterations, or significant behavioral changes; however, consistent increases in heart rate were observed, indicating a sublethal physiological response in the absence of overt developmental toxicity. In contrast, exposure under unadjusted conditions resulted in concentration-dependent increases in exposure pH and produced a coherent pattern of adverse effects, including reduced survival in acute assays and pronounced alterations in larval locomotor behavior, despite preserved morphology and high survival under sublethal conditions.

The present findings suggest that exposure-medium pH influences the apparent toxicity profile of DEA, particularly for physiological and behavioral endpoints, although the contributions of chemical exposure and medium alkalinity could not be fully separated. Collectively, these findings highlight the importance of considering exposure-medium pH when assessing the toxicity of alkaline compounds such as DEA, as pH changes associated with chemical exposure may contribute to the observed biological responses. They also support the use of zebrafish early life stages as an informative model for investigating sublethal effects of industrial chemicals. From an ecotoxicological perspective, the effects observed under unadjusted conditions highlight potential hazards associated with DEA-containing effluents, although their environmental relevance depends on DEA concentrations and the physicochemical conditions of receiving waters. Future studies incorporating molecular and biochemical approaches may further contribute to elucidating the mechanisms underlying DEA-induced toxicity.

## Figures and Tables

**Table 1:** pH of DEA-Unadj exposure media measured before and after each 24-h renewal during the LC_50_ experiments. Values are presented as mean ± SD from 4 independent experiments.

Group (mg/L)	pH Pre-Exposure (Mean ± SD)	pH Post-Exposure (Mean ± SD)
Control	7.52 ± 0.15	7.45 ± 0.16
300	9.51 ± 0.10	8.47 ± 0.10
500	9.66 ± 0.11	8.68 ± 0.10
700	9.74 ± 0.16	8.82 ± 0.10
900	9.83 ± 0.15	8.92 ± 0.06
1100	9.88 ± 0.12	9.01 ± 0.06
1300	9.92 ± 0.07	9.01 ± 0.07
1600	9.96 ± 0.08	9.09 ± 0.20
2000	10.00 ± 0.07	9.15 ± 0.24
2400	10.10 ± 0.07	9.12 ± 0.07
2800	10.10 ± 0.07	9.18 ± 0.04

**Table 2:** pH of DEA-Unadj exposure media measured before and after each 24-h renewal during the sublethal experiments. Values are presented as mean ± SD from 4 independent experiments.

Group (mg/L)	pH Pre-Exposure (Mean ± SD)	pH Post-Exposure (Mean ± SD)
Control	7.15 ± 0.36	6.99 ± 0.40
10	8.18 ± 0.18	7.21 ± 0.33
30	8.76 ± 0.10	7.43 ± 0.26
75	9.07 ± 0.11	7.71 ± 0.21
150	9.24 ± 0.13	8.01 ± 0.16
300	9.36 ± 0.15	8.30 ± 0.10

## Data Availability

The datasets generated during and/or analyzed during the current study are available in the repository Zenodo at https://doi.org/10.5281/zenodo.23038775.

## Supplementary Materials

The following supporting information can be downloaded at https://www.mdpi.com/article/10.3390/ijms27219935/s1.

## Abbreviations

The following abbreviations are used in this manuscript:

CO_2_	Carbon dioxide
DEA	Diethanolamine
DEA-Adj	Diethanolamine under pH-adjusted
DEA-Unadj	Diethanolamine under pH-unadjusted
FET	Fish embryo toxicity
hpf	hours post-fertilization
LC_50_	Median lethal concentration
OECD	Organization for Economic Co-operation and Development
SEM	Standard error of the mean
STC	Spontaneous tail coiling
