# Supplementary material for: pH-Dependent Developmental and Behavioral Toxicity of Diethanolamine in Zebrafish (Danio rerio)
Source: Int J Mol Sci. 2026 Nov 6;27(21):9935. doi: 10.3390/ijms27219935 (PMC13764351; doi:10.3390/ijms27219935)
Supplement: Supplementary file 1 [file ijms-27-09935-s001.zip › ijms-4644278-supplementary.pdf]

Supplementary material

Table S1. pH of DEA-Adj exposure media measured before and after each 24-h renewal during LC_{50} experiments. Values are presented as mean ± SD from 2 independent experiments

Group (mg/L)	pH pre-exposure (mean ± SD)	pH post-exposure (mean ± SD)
Control	7.36 ± 0.07	7.42 ± 0.11
125	7.47 ± 0.09	7.58 ± 0.12
250	7.45 ± 0.08	7.64 ± 0.14
500	7.41 ± 0.08	7.63 ± 0.16
1000	7.38 ± 0.08	7.56 ± 0.12
2000	7.35 ± 0.07	7.49 ± 0.08

Table S2. pH of DEA-Adj exposure media measured before and after each 24-h renewal during sublethal experiments. Values are presented as mean ± SD from 2 independent experiments

Group (mg/L)	pH pre-exposure (mean ± SD)	pH post-exposure (mean ± SD)
Control	7.24 ± 0.03	7.15 ± 0.11
50	7.35 ± 0.16	7.34 ± 0.14
250	7.44 ± 0.05	7.53 ± 0.09
500	7.45 ± 0.03	7.58 ± 0.02
1000	7.40 ± 0.10	7.57 ± 0.01
2000	7.44 ± 0.02	7.53 ± 0.01

Table S3. Conductivity of DEA-Adj exposure media measured before and after each 24-h renewal during LC_{50} experiments. Values are presented as mean ± SD from 2 independent experiments

Group (mg/L)	Conductivity pre-exposure (µS/cm; mean ± SD)	Conductivity post-exposure (µS/cm; mean ± SD)
Control	513.5 ± 42.6	513.9 ± 41.8
125	610.1 ± 52.14	627.4 ± 39.05
250	754.6 ± 39.2	772.3 ± 51.71
500	977.3 ± 38.54	985.5 ± 35.64
1000	1406 ± 45.87	1420 ± 38.98
2000	2000 ± 0.04	2000 ± 0.05

Table S4. Conductivity of DEA-Adj exposure media measured before and after each 24-h renewal during sublethal experiments. Values are presented as mean ± SD from 2 independent experiments

Group (mg/L)	Conductivity pre-exposure (µS/cm; mean ± SD)	Conductivity post-exposure (µS/cm; mean ± SD)
Control	460.6 ± 4.8	483.8 ± 18.9
50	520.2 ± 19.0	526.5 ± 4.7
250	663.0 ± 15.5	704.0 ± 9.2
500	881.2 ± 17.6	919.8 ± 12.1
1000	1259.0 ± 38.4	1356 ± 28.1
2000	2000.0 ± 0.5	2000 ± 0.03

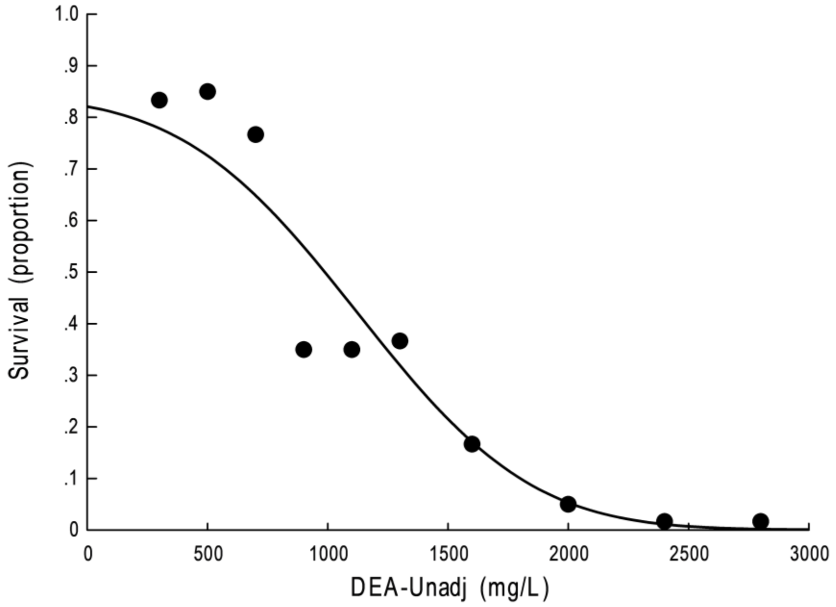

Figure S1. Concentration–response curve for zebrafish embryo survival after 96 hours exposure to DEA-Unadj, conducted according to the OECD Fish Embryo Acute Toxicity (FET) test guideline (OECD 236). Points represent mean survival proportion per concentration (*n* = 60-80 embryos per concentration, from three to four independent experiments), and the solid line represents the fitted concentration–response curve. The estimated LC_{50} value for DEA-Unadj was 1123.8 mg/L (95% CI: 1034.3-1213.2 mg/L), calculated using the Toxicity Relationship Analysis Program (TRAP, v1.30; U.S. EPA).

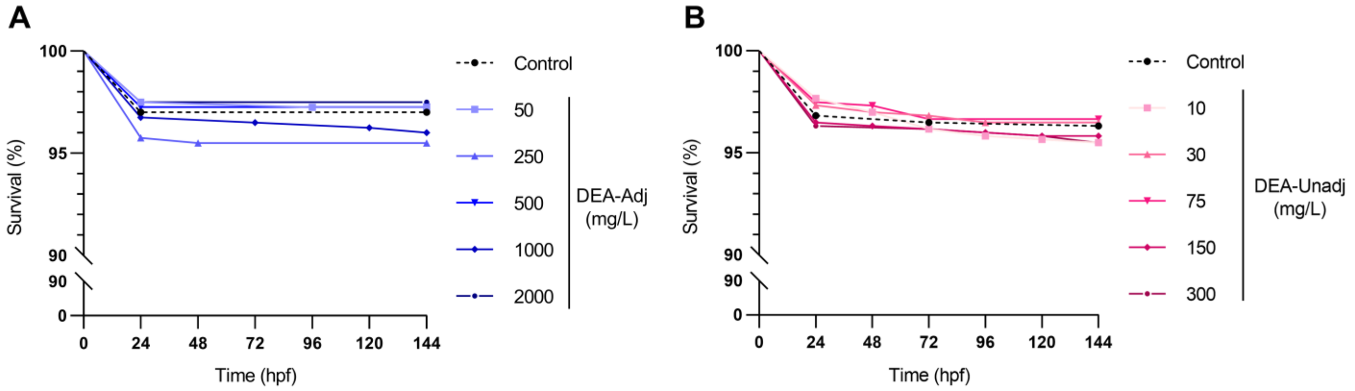

Figure S2. Survival of zebrafish animals exposed to diethanolamine with pH adjustment (DEA-Adj) and without pH adjustment (DEA-Unadj) from 3 to 144 hpf. (**A**) DEA-Adj groups compared with the control. (**B**) DEA-Unadj groups compared with the control. Data are expressed as percentage survival over time. For DEA-Adj, data represents four independent experiments, with one Petri dish per group per experiment (*n* = 4, 100 embryos/larvae per Petri dish per group; 400 embryos/larvae assessed per group); for DEA-Unadj, data represents five independent experiments, with one Petri dish per group per experiment (*n* = 5, 120 embryos/larvae per Petri dish per group; 600 embryos/larvae assessed per group). Survival was analyzed using the Log-Rank (Mantel–Cox) test.

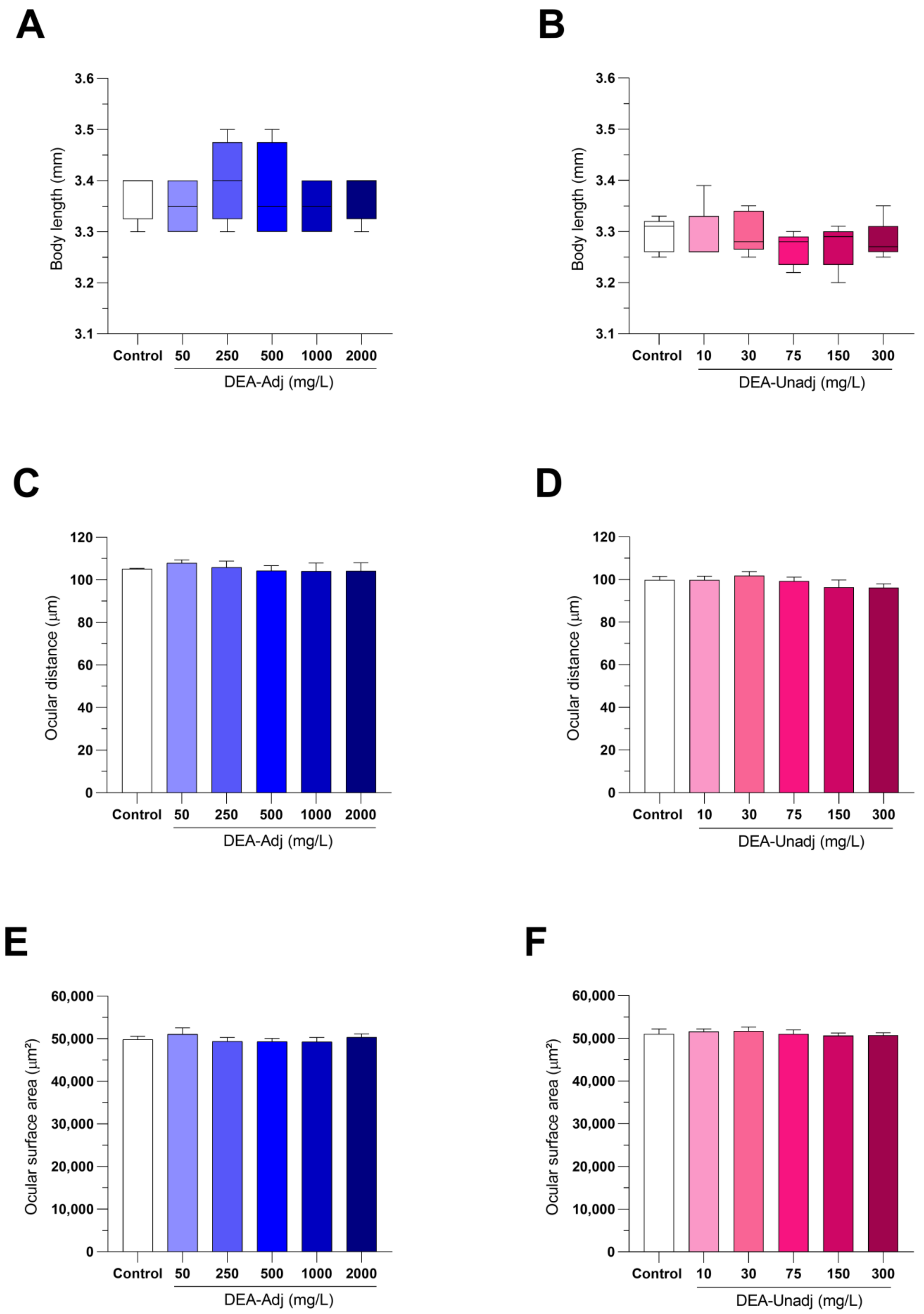
A
Body length (mm)
Control
50
250
500
1000
2000
DEA-Adj (mg/L)
B
Body length (mm)
Control
10
30
75
150
300
DEA-Unadj (mg/L)
C
Ocular distance (µm)
D
Ocular distance (µm)
E
Ocular surface area (µm²)
F
Ocular surface area (µm²)

Figure S3. Effects of diethanolamine (DEA) exposure on zebrafish larval morphology at 120 hpf. (**A**, **B**) Body length. Box-and-whisker plots represent the distribution of vessel-level means, with whiskers indicating the minimum and maximum values. Data distribution was assessed using the Shapiro–Wilk test, followed by Kruskal–Wallis followed by Dunn's post-hoc test. (**C**,**D**) Ocular distance and (**E**,**F**) Ocular surface area. Column bars represent mean ± standard deviation. Data distribution was assessed using the Shapiro–Wilk test, followed by one-way ANOVA and Dunnett's post hoc test for multiple comparisons against the control group. For DEA-Adj, data represents four independent experiments, with one vessel per group per experiment ($n = 4$, pool of 6 larvae per group); for DEA-Adj, data represents five independent experiments, with one vessel per group per experiment ($n = 5$, pool of 6 larvae per group).

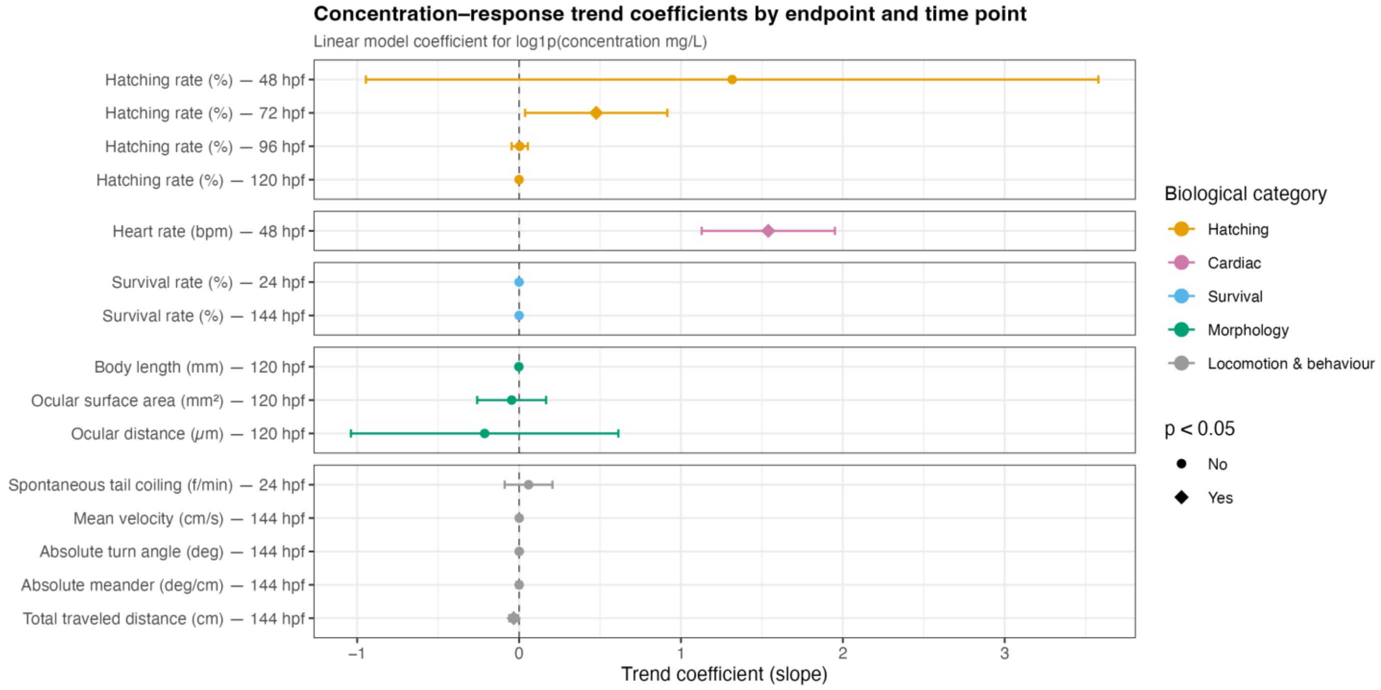

Figure S4. DEA-Adj concentration–response trend coefficients across all endpoints and time points. Each point represents the slope coefficient from a linear model fitted to vessel-level values, with log1p-transformed concentration (log1p[mg/L]) as a continuous predictor and experiment as a fixed blocking factor. Error bars indicate 95% confidence intervals. Points are colored by biological category: hatching, cardiac, survival, morphology, locomotion, and behavior. Positive coefficients indicate increasing endpoint values with increasing DEA concentration; negative coefficients indicate decreasing values.

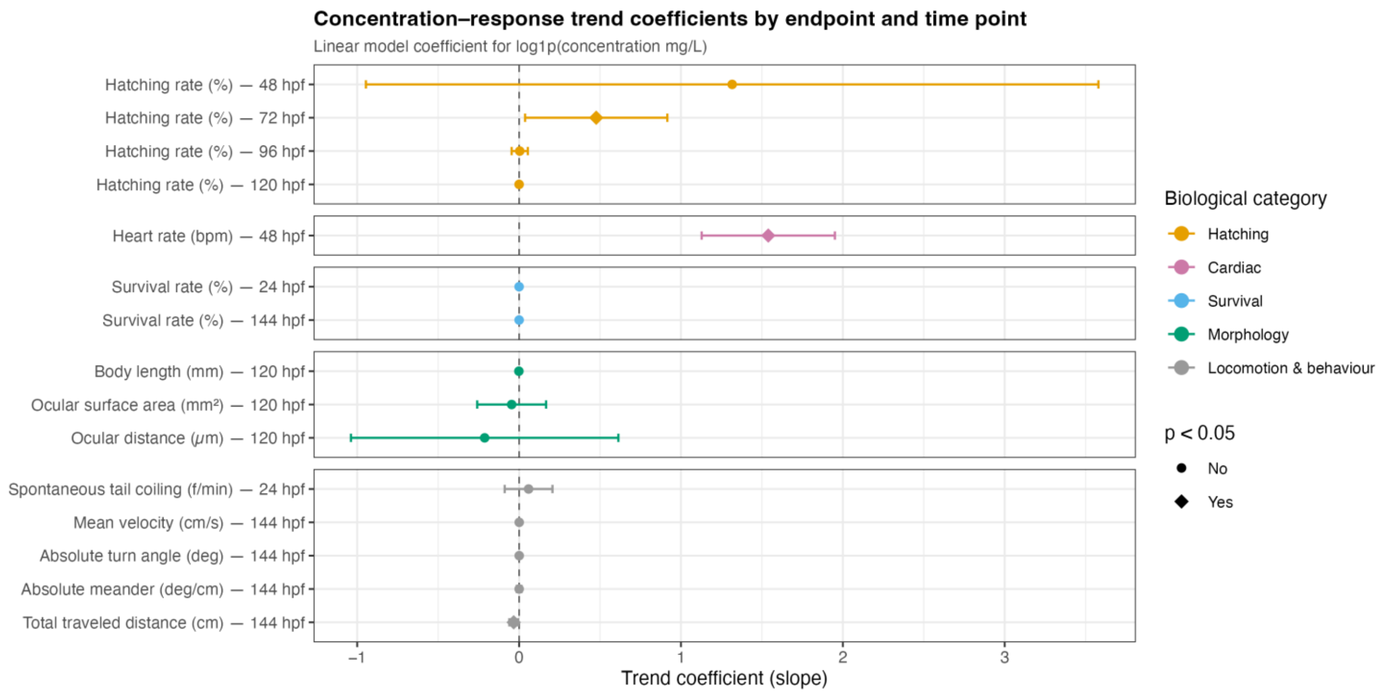

Figure S5. DEA-Unadj concentration–response trend coefficients across all endpoints and time points. Each point represents the slope coefficient from a linear model fitted to vessel-level values, with log1p-transformed concentration (log1p[mg/L]) as a continuous predictor and experiment as a fixed blocking factor. Error bars indicate 95% confidence intervals. Points are colored by biological category: hatching, cardiac, survival, morphology, locomotion, and behavior. Positive coefficients indicate increasing endpoint values with increasing DEA concentration; negative coefficients indicate decreasing values.

Abbreviations

The following abbreviations are used in this manuscript:

CO_2	Carbon dioxide
DEA	Diethanolamine
DEA-Adj	Diethanolamine under pH-adjusted
DEA-Unadj	Diethanolamine under pH-unadjusted
FET	Fish embryo toxicity
hpf	hours post-fertilization
LC_{50}	Median lethal concentration
OECD	Organization for Economic Co-operation and Development
SEM	Standard error of the mean
SD	Standard deviation
